# What factors affect the severity of permanent tooth impaction?

**DOI:** 10.1186/s12903-018-0649-5

**Published:** 2018-11-01

**Authors:** Mariam Al-Abdallah, Abeer AlHadidi, Mohammad Hammad, Najla Dar-Odeh

**Affiliations:** 1 Department of Orthodontics, Faculty of Dentistry, The University of Jordan, Amman, 11942 Jordan; 2 Department of Oral and Maxillofacial Surgery, School of Dentistry, The University of Jordan, Amman, Jordan; 3 Department of Conservative Dentistry, Faculty of Dentistry, The University of Jordan, Amman, Jordan; 4 Department of Oral Medicine, Faculty of Dentistry, The University of Jordan, Amman, Jordan

**Keywords:** Impaction, Dental anomalies, Malocclusion, Severity

## Abstract

**Background:**

The aim of this study was to investigate the association between the severity of permanent tooth impaction and a number of predefined factors, including tooth type, age, gender, tooth agenesis, microdontia of maxillary lateral incisor, and retained deciduous predecessors.

**Methods:**

A sample of 2979 dental patients, aged 15 to 40 years, was surveyed by two calibrated examiners for permanent tooth impaction (excluding third molars). On panoramic radiographs, the impacted teeth were initially ranked based on their vertical, horizontal, and angular positions, and the ranking was then analysed for distribution by the predefined factors. To test the age factor, patients were divided into younger (15 to 25 yr) and older (between 25 and 40 yr) age groups. The statistical significance of the ranked vertical, horizontal, and angular positions of impacted teeth by the investigated factors was determined using the Wilcoxon-Mann-Whitney U test.

**Results:**

The angular position of the impacted teeth was more severe in the older age group (*P* = 0.012) and in females (*P* = 0.018). The maxillary canine had more severe horizontal (*P* = 0.001) and angular (*P* = 0.003) impactions in females. Tooth agenesis was associated with less severe horizontal impaction (*P* = 0.041) in the mandibular second premolar. In addition, microdontia of the maxillary lateral incisor was associated with more severe horizontal impaction in general, and more severe horizontal (*P* = 0.024) and angular (*P* = 0.010) impaction of the mandibular second premolar in particular. Finally, our results showed that a retained deciduous predecessor was linked to a less severe vertical impaction of the mandibular second premolar (*P* = 0.030) and horizontal impaction of the maxillary second premolar (*P* = 0.037) but more severe angular impaction of the mandibular canine.

**Conclusions:**

This study suggests that the more delayed the treatment, being a female, the presence of maxillary lateral incisor with microdontia, and retained lower deciduous canines might be associated with more severe position of the impacted teeth. Because the severity of tooth impaction would follow different patterns when considering the investigated factors, it is mandatory to include such factors during dental diagnoses and the planning of preventive or interceptive interventions for young patients.

## Background

The normal development of the occlusion and craniofacial complex is largely dependent on the normal physiological eruption of teeth [1]. Eruption is the process by which a tooth moves axially from its follicle position in the bone into its final functional position in the oral cavity. Following clinical and radiographic assessment, if a tooth is not expected to erupt, as a result of a positional deviation of its developing follicle or the presence of a physical barrier in its path, then the tooth is rendered impacted [1–4].

The impaction of permanent teeth (excluding third molars) is a frequent phenomenon, with a reported prevalence ranging from 2.9% [5] to 13.7% [5–13]. The most frequently impacted teeth are the canines and second premolars in both jaws with different incidence rates [6–16].

The orthodontic alignment of an impacted tooth to its normal functioning position in the oral cavity may require prolonged and complicated treatment. Prognosis and treatment difficulty can be affected by many factors, some of which are related to the patient, others to features associated with malocclusion, and most importantly, the factors related to the position of the impacted tooth [14–18].

As dentists, we are interested in the prevalence and pattern of impacted teeth, as well as identifying the factors that might affect the severity of the impaction and, consequently, the difficultly and duration of treatment needed [14, 19, 20]. Most previous studies have focused on the prevalence of impacted teeth and described their position in the jaws. Therefore, the aim of the present study is to investigate factors affecting the severity of the impaction of permanent teeth.

## Methods

Digital panoramic radiographs and clinical records for 4258 dental patients aged 15 to 40 years old who attended a university dental hospital between 2011 and 2015 were retrieved for this study. All digital panoramic radiographs were taken with the KODAK 8000 Digital Panoramic System® and viewed using the KODAK Dental Imaging Software®. Patients with incomplete clinical records, craniofacial syndromes and clefts, or previous history of extraction or orthodontic treatments were excluded. Following these criteria, 2979 cases were included in this study (average age = 24.9 years, males = 45.3%, females = 54.7%).

Two calibrated researchers (M.A. and A.A.) examined the selected records concurrently to determine the presence, tooth involved and the position of impaction of permanent teeth (excluding third molars). A tooth was diagnosed as impacted when it was predicted to remain unerupted due to a physical barrier or deflection along its eruption path, or if it remained in the jaw 2 years past the expected eruption time [2, 6, 21, 22]. The minimum age for inclusion was 15 years to account for the delayed development and eruption of second premolars, thereby minimizing false-positive findings [23].

The following variables were recorded to describe the vertical, horizontal, and angular positions of each impacted tooth:

1. Vertical position: The distance of the crown of the impacted tooth from the occlusal plane was ranked relative to the adjacent mesial tooth. Horizontal lines were drawn and vertical sectors were formed to locate the impacted tooth vertically [14, 24]. The ranking of the used sectors were as follows:
  Rank 1: Occlusal to the cemento-enamel junction.
  Rank 2: Within the occlusal half of the root.
  Rank 3: Within the apical half of the root.
  Rank 4: In a position more apical than the apex.
2. Horizontal position: Judged relative to the vertical lines dividing the area adjacent to the impacted tooth [16]. The position of the impacted tooth was then determined relative to the formed sectors. The sectors used were ranked as follows:
  Rank 0: Absence of horizontal overlap.
  Rank 1: Overlap with the distal half of the root of the tooth mesial to the impacted tooth.
  Rank 2: Overlap reaching the mesial half of the root of the tooth mesial to the impacted tooth.
  Rank 3: Overlap reaching the distal half of the root of the second tooth mesial to the impacted tooth.
  Rank4: Overlap reaching the mesial half of the second tooth mesial to the impacted tooth.
  Rank 5: Overlap beyond two adjacent teeth mesial to the impacted tooth.
3. Angular position: Assessed by measuring the angle between the midline and the long axis of the impacted tooth [14–16]. The angle was then ranked as follows:
  Rank 1: less than 30 degrees
  Rank 2: 30–45 degrees
  Rank 3: more than 45 degrees

Data ranking the position of the impacted teeth were pooled and analysed for distribution by age and gender, as well as association with dental anomalies. For the purpose of investigating the significant effects of age on the severity of impacted tooth position, the sample was divided into two groups: adolescents and young adults with an age ranging from 15 to 25 years (the younger age group), and adults between the ages of 25 and 40 (the older age group).

The dental anomalies included in this investigation were:

1. Tooth agenesis: no sign of crown calcification on the radiograph and no evidence or history of loss attributable to caries, periodontal disease, or trauma [25, 26].
2. Microdontia of maxillary lateral incisor: mesio-distal width of the crown less than that of the opposing mandibular lateral incisor [27].
3. Retained deciduous tooth: deciduous tooth maintained in the arch with the presence of an impacted permanent successor.

The Statistical Package for Social Sciences (SPSS software for Windows, version 22, Chicago, Illinois) was used for the statistical analysis. The prevalence of impacted teeth in both age groups and genders were compared using a chi-square test. On the other hand, an independent sample t-test was used to compare the number of impacted teeth between the age and gender groups. The Wilcoxon-Mann-Whitney U test was conducted to determine the statistical significance of the ranked vertical, horizontal, and angular positions of the impacted teeth by tooth type, age, gender, tooth agenesis, microdontia of upper lateral incisor, and retained deciduous teeth. The level of significance for all tests was set at *P* < 0.05.

## Results

**Fig. 1:**
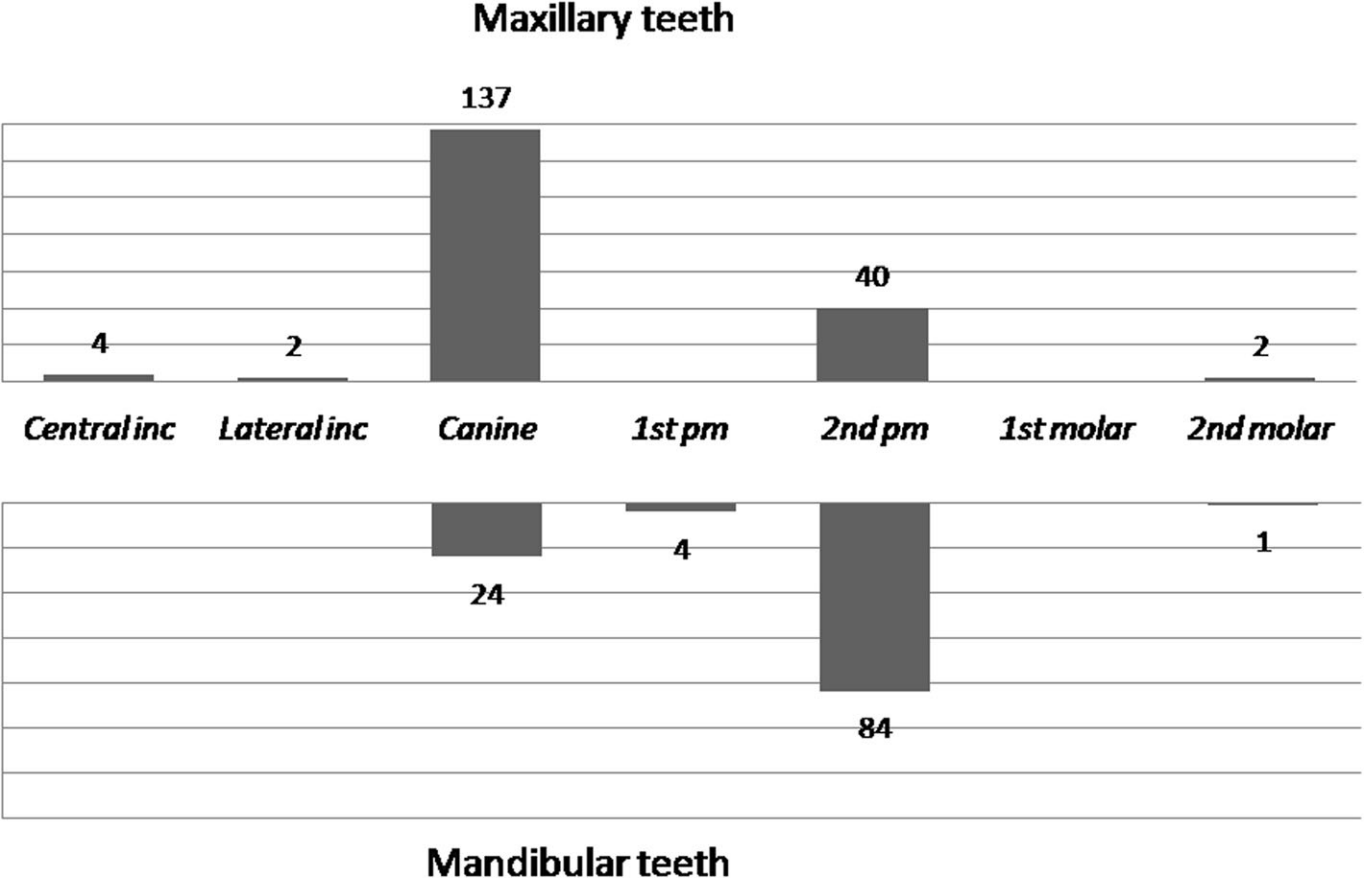
Distribution of the 297 impacted teeth between the maxilla and the mandible. Inc.: incisor, 1st pm: first premolar, 2nd pm: second premolar

From the total of 2979 selected and analysed records, at least one impacted tooth was diagnosed in 189 dental patients (males = 46.6%, females = 53.4%, mean age = 23.4 yr., SD = 7.1). The prevalence of impaction in the younger age group (*n* = 123) was 6.8%, and in the older age group (*n* = 66), the prevalence was 5.6%; there were no significant differences in the prevalence between the two age groups (X^2^ = 1.703, *P* = 0.19). The total number of impacted teeth was 297, with an average of 1.6 impacted teeth per patient. The distribution of the 297 impacted teeth between the maxilla and the mandible is shown in Fig. 1. The four most frequently impacted teeth were the maxillary canine (46.1%), the mandibular second premolar (28.2%), the maxillary second premolar (13.5%), and the mandibular canine (8.1%).

Following the ranking of the position of the impacted teeth in the vertical, horizontal and angular categories, statistical significance testing was carried out to determine if any of the investigated factors affected the severity of impaction (Table 1). When the older age group was compared to the younger age group, there was worsening in the position of the impaction in all three categories with age, but it did not reach a significant level, except for the angle of impaction (*P* = 0.012).

**Table 1:** Severity of permanent tooth impaction in the vertical, horizontal, and angular positions in relation to different grouping factors. (younger age group = 15-25 yr., older age group = 25.1-40 yr., Mx2 = maxillary lateral incisor)

Position of the impacted tooth (*n* = 297)	Factors assessed in relation to the severity of impaction using Wilcoxon-Mann-Whitney test (*P*-value)														
	Age			Gender			Tooth agenesis			Microdontia of Mx2			Retained deciduous		
	Younger Mean rank	Older Mean rank	*P*-value	Male Mean rank	Female Mean rank	*P*-value	Yes Mean rank	No Mean rank	*P*-value	Yes Mean rank	No Mean rank	*P*-value	Yes Mean rank	No Mean rank	*P*-value
Vertical	129.2	132.6	0.675	130.8	130.3	0.949	126.1	131.2	0.651	141.0	128.8	0.279	130.3	130.7	0.961
Horizontal	147.0	152.5	0.573	140.5	156.8	0.087	138.7	150.6	0.398	175.4	145.0	0.030*	156.8	143.1	0.150
Angle of impaction	140.5	163.9	0.012*	137.9	159.1	0.018*	139.9	150.4	0.430	168.6	146.0	0.088	154.7	144.7	0.267

* : *P*<0.05 (statistically significant difference)

Males and females had comparable rankings for the vertical and horizontal impactions. Nevertheless, the angle of impaction was more severe in females compared to males (*P* = 0.018). On the other hand, ranking of the position of the impacted teeth was lower for patients with tooth agenesis when compared to those with full permanent dentition, but the values were not statistically significant. Microdontia of the maxillary lateral incisor was associated with a higher ranking of the position of the impaction, but again, the values were not significant, except for the horizontal impaction (*P* = 0.030). Although the horizontal impaction of teeth with a retained predecessor was more severe, none of the values were statistically significant.

When the ranking of the position of the impaction of the four most prevalent impacted teeth were compared, it was found that the maxillary canine was the most severely impacted in the vertical, horizontal and angular positions (*P* < 0.001), while the mandibular second premolar was the least severely impacted (*P* < 0.001). The severity of impaction for these four teeth was assessed by age, gender and associated dental anomalies, as shown in Table 2.

**Table 2:** Severity of permanent tooth impaction in the vertical, horizontal, and angular positions in relation to different grouping factors. (younger age group = 15-25 yr., older age group = 25.1-40 yr., Mx2 = maxillary lateral incisor, V = vertical, H = horizontal, A = angle of impaction)

Impacted tooth under investigation	Impaction position	Factors assessed in relation to the severity of impaction using Wilcoxon-Mann-Whitney test (*P* value)														
		Age			Gender			Tooth agenesis			Microdontia of Mx2			Retained deciduous		
		Younger Mean rank	Older Mean rank	*P* value	Male Mean rank	Female Mean rank	*P* value	Yes Mean rank	No Mean rank	*P* value	Yes Mean rank	No Mean rank	*P* value	Yes Mean rank	No Mean rank	*P* value
Maxillary canine (*n* = 137)	V	63.4	67.4	0.494	65.8	64.3	0.804	69.6	64.3	0.550	67.2	64.6	0.737	65.3	64.7	0.921
	H	64.2	76.1	0.080	57.9	79.0	0.001*	62.2	70.0	0.443	76.4	67.7	0.344	68.1	69.9	0.792
	A	62.5	78.7	0.012*	59.0	78.0	0.003*	62.6	69.9	0.451	71.3	68.6	0.759	66.0	72.1	0.333
Mandibular 2nd premolar (*n* = 84)	V	32.8	34.7	0.531	33.7	33.3	0.896	36.0	33.3	0.574	36.0	33.1	0.453	29.6	35.9	0.030*
	H	43.4	40.5	0.553	44.4	40.9	0.437	28.1	44.0	0.041*	55.1	40.4	0.024*	42.3	42.6	0.956
	A	40.3	47.1	0.057	42.6	42.4	0.937	36.0	43.2	0.207	53.1	40.7	0.010*	40.2	43.7	0.326
Maxillary 2nd premolar (*n* = 40)	V	16.7	16.0	0.765	16.7	16.4	0.890	14.7	17.2	0.309	20.5	15.8	0.127	14.6	19.0	0.054
	H	22.4	16.6	0.114	23.3	17.9	0.117	24.3	19.4	0.231	21.2	20.4	0.878	17.3	24.5	0.037*
	A	20.1	21.2	0.740	18.4	22.4	0.184	22.5	19.9	0.481	23.6	20.1	0.443	20.7	20.3	0.908
Mandibular canine (*n* = 24)	V	11.3	10.7	0.812	10.0	12.1	0.410	8.5	11.6	0.318	0	0	–	12.1	10.6	0.573
	H	13.4	11.0	0.394	12.8	12.2	0.831	10.9	12.8	0.594	0	0	–	16.4	11.2	0.097
	A	13.0	11.7	0.602	13.1	11.9	0.613	9.0	13.2	0.175	0	0	–	16.6	11.1	0.041*

* : *P*<0.05 (statistically significant difference)

Both age groups had comparable severity of impaction of all teeth except for the maxillary canine. The older age group had a significantly more severe angle of impaction (*P* = 0.012) of the maxillary canine compared to the younger age group. Correspondingly, males and females had a similar severity of impaction in all teeth except for the maxillary canine, which had more severe impactions in females in the horizontal (*P* = 0.001) and angular (*P* = 0.003) positions.

Tooth agenesis did not affect the severity of impaction, except for in the mandibular second premolar where the horizontal impaction position was less severe (*P* = 0.041). Similarly, the microdontia of the maxillary lateral incisor only significantly affected the horizontal (*P* = 0.024) and angular (*P* = 0.010) positions of the impacted mandibular second premolar, but it was more severe. A retained deciduous predecessor was linked to a reduced severity of the vertical impaction of the mandibular second premolar (*P* = 0.030), a reduced severity of the horizontal impaction of the maxillary second premolar (*P* = 0.037), and an increased severity of the angular impaction of the mandibular canine (*P* = 0.041). Nevertheless, a retained deciduous predecessor had no effect on the severity of the impaction of the maxillary canine.

## Discussion

An unerupted tooth is considered a clinical challenge for the orthodontist in terms of diagnosis, anchorage management and treatment duration. A treatment difficulty index published by Pitt et al. in 2006 has been used to predict and evaluate such a challenge [14]. Part of the difficulty score used in this index is based on ranking the position of the impacted teeth. The higher the rank of the position of the impacted tooth, the more difficult it is to align [15, 20, 24]. In our study, we investigated the effects of a number of factors on the severity of impaction. The results showed that different tooth types vary significantly in the severity of impaction and, consequently, in the difficulty of treatment. Among the four most frequently impacted teeth found in our study, the maxillary canine was the most severely impacted (*P* < 0.001), whereas the mandibular second premolar was the least severely impacted (*P* < 0.001). Therefore, tooth type could be another factor that must be considered to predict treatment difficulty of impacted teeth.

The second factor affecting the severity of tooth impaction that was investigated in our study was age. The results suggested that, as time passed, there might be a risk of the position of the impacted tooth worsening, especially increasing the angle of its long axis towards the midline (*P* = 0.012). Therefore, the earlier the diagnosis and treatment of the impacted tooth, the less complicated and shorter the treatment duration will be, as suggested by the treatment difficulty index [14]. The angular impaction of the maxillary canine was significantly worse in the older age group (*P* = 0.012). This finding might suggest that a severely impacted tooth can migrate and cross the midline with time, which stresses the importance of an early diagnosis and treatment planning.

There might have been differences between genders when the prevalence of dental impaction was investigated [11, 13]. Nevertheless, none of the previous studies reported the effect of gender on the severity of impaction of permanent teeth. In the current study, the Wilcoxon-Mann-Whitney test showed a significantly more severe angular tooth impaction in females compared to males (*P* = 0.018). When tooth type was taken into consideration, the maxillary canine had worse horizontal (*P* = 0.001) and angular (*P* = 0.003) impactions in females. This finding might indicate x-linked genetic factors contribute to the aetiology and prognosis of maxillary canine impactions. It also suggests that when maxillary canine impaction is diagnosed in females, it is predicted to be more severe, and therefore, a more difficult and lengthy treatment is to be expected. Furthermore, permanent teeth erupt earlier in females; therefore, early inspection and palpation of maxillary canines and early interceptive procedures [16] should be considered crucial clinical routines in female patients.

Tooth agenesis was the next factor investigated for its effect on the severity of tooth impaction in the present study. Tooth agenesis was proven to be significantly associated with a higher incidence of impaction of permanent teeth [25, 27–29]; however, only a few studies have investigated the effects of this factor on the severity of impaction [23, 30, 31]. Our results showed that tooth agenesis was associated with a reduced severity of impaction of all teeth in general, but not on a significant level. Only the mandibular second premolar had a significant reduction in the severity of the horizontal impaction (*P* = 0.041) when tooth agenesis was present.

Unlike tooth agenesis, microdontia of the maxillary lateral incisor was associated with more severe impaction, especially in the horizontal position (*P* = 0.030). Surprisingly, the most severely affected tooth was not the maxillary canine, as expected [32, 33]; it was the mandibular second premolar, especially in the horizontal (*P* = 0.024) and angular (*P* = 0.010) positions. A genetic link between dental anomalies affecting the maxillary lateral incisor and the mandibular second premolar was suggested by previous studies [27, 29, 34]; however, none of those studies looked specifically on the severity of impaction. Similarly, retained deciduous teeth were not researched previously for their effects on the severity of the impaction of permanent teeth. The findings of the present study suggest that maintaining the deciduous second molar might be recommended, as its presence was linked to a reduced severity of the vertical impaction of the mandibular second premolar (*P* = 0.030) and the horizontal impaction of the maxillary second premolar (*P* = 0.037). On the other hand, our results emphasize the importance of timely extraction of the mandibular deciduous canine if the permanent successor shows risk of impaction, because a retained deciduous tooth was significantly associated with an increased severity of the angular impaction (*P* = 0.041) of the mandibular canine.

## Conclusions

1. With age, the angle of an impacted tooth might increase in severity; therefore, early diagnosis and treatment is mandatory, especially for the maxillary canines.
2. Females suffer from more severe impaction of teeth in general, and of the maxillary canine in particular. Consequently, and because teeth erupt earlier in females, it is crucial to diagnose impaction earlier in females and carryout any necessary preventive or interceptive orthodontic procedures.
3. The presence of microdontia of the maxillary lateral incisor is significantly associated with more severe impaction, which emphasizes the importance of tooth size investigations in young patients and carrying out further analysis for those with small laterals.
4. Finally, the results of the current study revealed a significant association between retained deciduous predecessors and the severity of impaction of their successors. Nevertheless, an observational longitudinal study is required to produce solid clinical recommendations.

## Abbreviations

A: Angle of impaction
H: Horizontal
Mx2: Maxillary lateral incisor
n: number
SD: Standard deviation
V: Vertical
X^2^: Chi-squared test
yr.: years
